# The role of adhesion protein Fibulin7 in development and diseases

**DOI:** 10.1186/s10020-020-00169-z

**Published:** 2020-05-19

**Authors:** Papiya Chakraborty, Shiba Prasad Dash, Pranita P. Sarangi

**Affiliations:** Department of Biotechnology, Indian Institute of Technology Roorkee, Roorkee, Uttarakhand 247667 India

**Keywords:** Fibulins, Fibulin-7, Extracellular matrix protein, Adhesion molecule, Therapeutics

## Abstract

Fibulins are a family of secreted glycoproteins, which play an important role in regulating multiple cellular functions such as adhesion, growth, motility, and survival. Fibulin7 (Fbln7) is expressed in developing odontoblasts, in the giant trophoblast layer of the placenta, in the choroid of the eyes as well as in the cartilage. Since its discovery, reports from various research groups have improved our understanding about the roles and effects of Fbln7 and Fbln7 derived fragments and peptides under physiological and pathological conditions such as tooth development, angiogenesis, immunoregulation, cancer pathogenesis and very recently as a possible biomarker for glaucoma. This review will highlight the latest developments in our understanding of the functions, the proposed mechanism of actions, and Fbln7’s possible implications in future research and as therapeutics for different diseases.

## Introduction

**Fig. 1:**
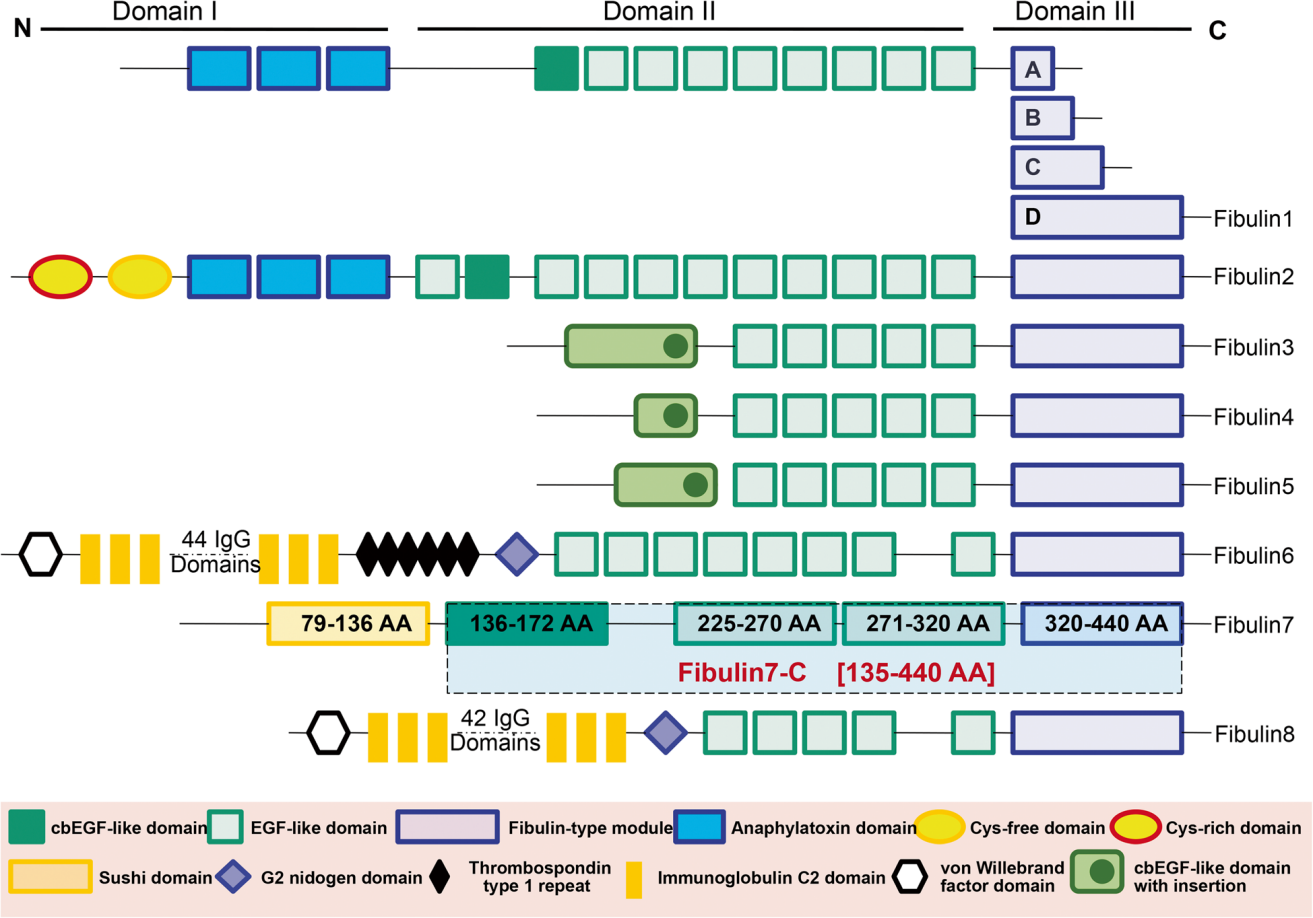
Domain structure of eight members of the fibulin family of proteins. The proteins are composed of three domains. Domain-I or the N-terminal domain varies among different fibulins, domain-II consists of a variable number of tandemly arranged epidermal growth factor (EGF)-like modules or calcium-binding EGF (cbEGF)-like modules while, domain-III or the C-terminal or fibulin-type module is unique to all fibulins. Fbln7 has an additional sushi domain on the N-terminal, and the shaded area in the C terminus shows the Fbln7-C fragment

Fibulins are a group of extracellular matrix glycoproteins, that are structurally characterized by a three-modular structure (de Vega et al. 2009; de Vega et al. 2007). Domain-I or the N-terminal domain varies among different fibulins, domain-II consists of a variable number of tandemly arranged epidermal growth factor (EGF)-like modules or calcium-binding EGF (cbEGF)-like modules while, domain-III or the C-terminal or fibulin-type module is unique to all fibulins (Fig. 1) (Giltay et al. 1999). The multi-modular structure of fibulins supports several interactions with other proteins, extracellular matrices, and growth factors and regulates various physiological functions such as growth, development, and cell adhesion. (de Vega et al. 2009; de Vega et al. 2007; Argraves et al. 1989; Auer-Grumbach et al. 2011a; Timpl et al. 2003). To date, 8 Fibulins have been identified (Fibulin 1–8), with all sharing a conserved Fibulin type C-terminal domain as described above. Several Fibulins such as Fibulin 1 and 2 also occur in multiple variants due to alternative splicing, which is expressed in different species or discrete tissues of the same species. For instance, splice variants C and D of Fibulin1 are present in mice, zebrafish, chickens, and nematodes (Barth et al. 1998), while variants A and B are present only in humans at low levels (Argraves et al. 1990).

Among the fibulins, Fibulin1 and Fibulin2, are characterized by the presence of an extra domain with three anaphylatoxin modules and additional calcium-binding cbEGF modules (Fig. 1). They bind to laminin alpha and gamma chains and regulate cellular functions such as adhesion, migration, and cell anchorage (Utani et al. 1997). Fibulin3 is found in developing bones, epithelial cells, and endothelial cells of certain parts of the body, while Fibulin4 and Fibulin5 are involved in elastic fiber formation (Zhang and Marmorstein 2010; Papke and Yanagisawa 2014). Studies have shown that fibulin4 knock-out mice die from rupture of aortic aneurysms during the perinatal period of development (Huang et al. 2010). Additionally, a recent study has shown that mutation in the *fibulin-5* gene also affects peripheral nerves, eyes, and skin causing age-related macular degeneration and neuropathies (Auer-Grumbach et al. 2011b). Fibulin6 and Fibulin8, also known as hemicentin-1 and -2 and are the largest members of the fibulin family with a molecular size of more than 600 kDa (Tsuda 2018; Xu et al. 2013). Mutational studies in the zebrafish model have shown the role of Fibulin6 in mesenchymal cell migration and epidermal-dermal junction formation. Fibulin8, on the other hand, has been shown to cooperate with fibulin1 during zebrafish development (Chowdhury et al. 2014; Feitosa et al. 2012). To date not much is known about these fibulins.

Fibulin7 (Fbln7) was first identified in the mouse tooth germ cDNA microarray by differential hybridization and was named TM14 (de Vega et al. 2007). It was identified as a protein consisting of 440 amino acids, containing a signal peptide, 3 EGF-modules, a C-terminal domain, and a unique N-terminal sushi domain. Sushi domain, which is also known as the complement control protein (CCP), is prevalent in the proteins involved in complement system regulation, blood coagulation, cell adhesion, migration, and embryogenesis (de Vega et al. 2007; Wharton et al. 1985; Williams and Barclay 1988; Furie and Furie 1988). Although not much is understood about the exact functions of Fbln7, deletion of critical regions in the human Fbln7 gene at 2q13 chromosome (2q13 deletion syndrome) in zebrafish, was found to be associated with multiple diseases and developmental anomalies (Russell et al. 2014). Additionally, its unique expression in specialized tissues such as the placenta, eye, blood vessels, and cartilage indicate an important role of Fbln7 in both physiological and pathological conditions. In an attempt to understand the functional relevance, de Vega et al., first prepared multiple fragments and tested them for a specific effect on angiogenesis. Since then, in less than a decade, many reports have highlighted the modulatory roles of the C-terminal fragment of Fbln7 protein (Fbln7-C) on the functions of Human Umbilical Vein Endothelial Cells (HUVEC) and leukocytes (de Vega et al. 2016; Sarangi et al. 2018; Ikeuchi et al. 2018). The following paragraphs will describe the recent reports on the functions of Fbln7 under physiological as well as pathological conditions.

### Tissue expression and possible function of Fbln7 in early development

**Fig. 2:**
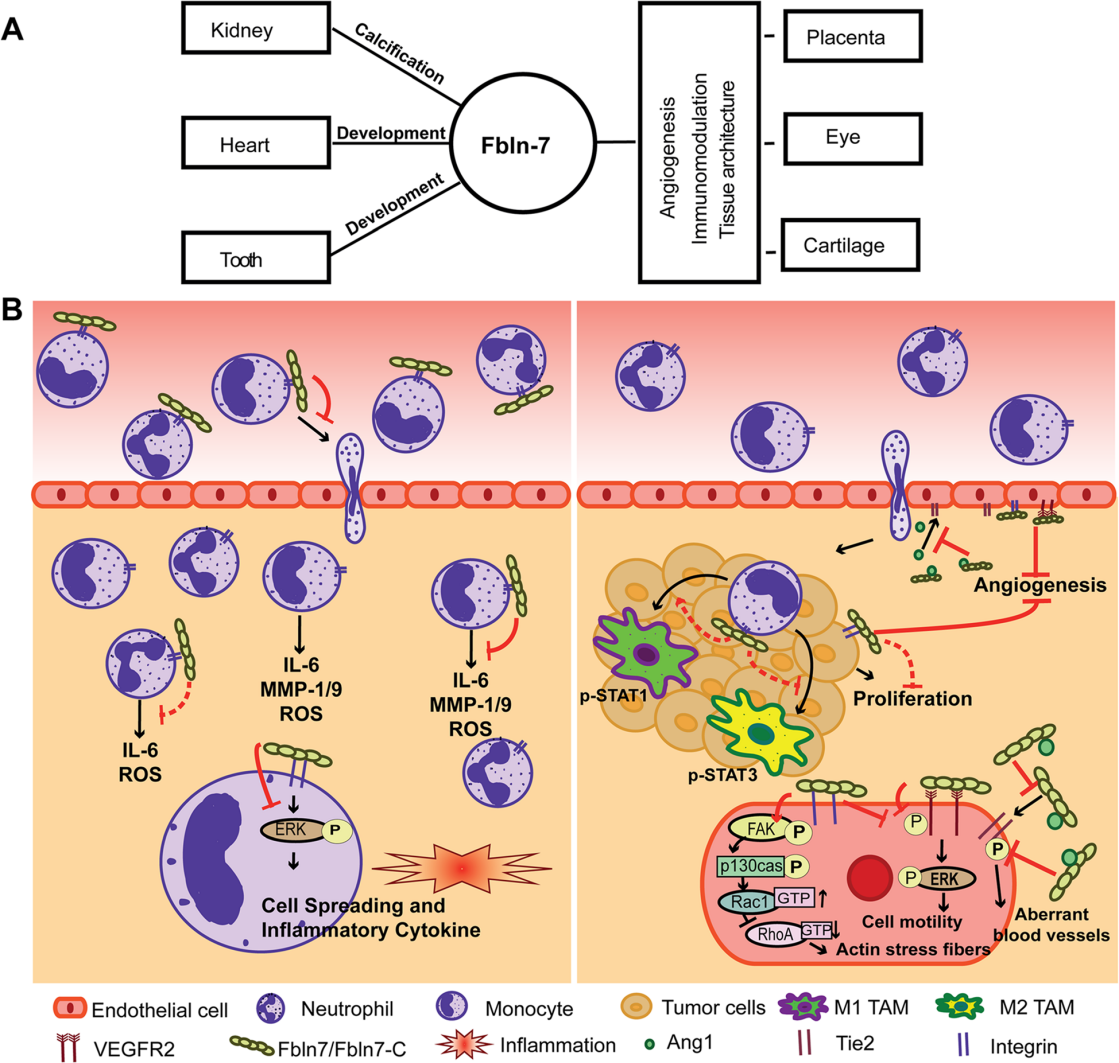
Role of Fbln7 and its C-terminal fragment in the modulation of cellular functions under physiological and pathological conditions. **a** Under physiological conditions, the Fbln7 protein plays an essential role in the modulation of blood vessel formation, development, cell adhesion in multiple tissues such as placenta, eye, and cartilages. **b** One of the C-terminal fragment of Fbln7, Fbln7-C binds to immune cells such as monocytes/macrophages and neutrophils via integrin β1 and regulate their migration and functions via binding to surface integrins and modulation of ERK1/2 and MAPK signaling pathway. Similarly, in tumor microenvironment interaction of cell surface integrin expressed on cancer cells, endothelium or TAMs inhibits the angiogenesis and progression of cancers via modulation of STAT and ERK pathways. Binding to VEGFR2 sustained activation of Rac1, and inhibition of VEGFR2 and ERK1/2 are possible mechanisms involved in the suppression of angiogenesis. Additionally, overexpression of Fbln7 also results in its binding to Ang1, thereby inhibiting the phosphorylation of Tie2 expressed in the endothelial cells

In the initial report on the identification of Fbln7, de Vega et al. showed that Fbln7 mRNA is expressed in developing teeth (de Vega et al. 2007). Precisely, Fbln7 is expressed highly in newborn incisors and molars, with comparatively weaker expression in kidneys, brain, muscles, and bones (de Vega et al. 2009; de Vega et al. 2007). Using in situ hybridization experiments with newborn molars, they observed that Fbln7 is highly expressed specifically in the odontoblasts that are formed from the differentiation of dental mesenchymal cells (de Vega et al. 2007). Their study suggested that Fbln7 that is co-localized with fibronectin, might act as an anchoring molecule for the attachment of odontoblasts to the dentine matrix. Besides, Fbln7 expression was also detected in the hair follicles and chondrocytes present in the proliferative zone, articular cartilage, spongiotrophoblast cells of E16 placenta including endothelial cells in blood vessels and choroid of the eye (Fig. 2a) (de Vega et al. 2007; de Vega et al. 2014). In a latest and interesting study, Tsunezumi et al. showed that, in contrast to the mild expression of Fbln7 in the kidney of newborn mice, in adult kidneys, they were highly expressed in the renal tubular epithelium, bowman’s capsule epithelium, and perivascular region. The study has shown that although the protein does not affect the metabolism of calcium and phosphate, it facilitates the local deposition of calcium phosphate crystals to renal tubular cells, thus promoting ectopic calcification in adult mice. However, the binding of heparin to the N terminal coiled-coil domain interspaced between 28 to 73 amino acid residues inhibits calcification, suggesting a regulatory role of Fbln7 in renal calcification. Higher expression of Fbln7 in adult mice depicts a significant role of this protein in development. Additionally, the expression of Fbln7 was also evident in the ureter and ovary of adult mice, whose functions are not explored yet. It would be interesting to study the functions of Fbln7 in the ovary, as other calcium-binding proteins such as S100A2 and S100A10 are associated with bad prognosis in ovarian cancers (Fig. 2a). This report supported the initial finding by de Vega et al., where they showed that Fbln7 interacts with other matrix proteins such as Fibronectin, Dentin sialophosphoprotein (DSPP), and Fibulin1 (de Vega et al. 2007; Tsunezumi et al. 2018). While the original paper indicated that kidneys express low levels of Fbln7 compared to incisors, the later paper showed more robust expression in the kidney with functional relevance.

Fbln7 is expressed in multiple vital tissues such as the eye, placenta, cartilage, bones, teeth, including blood vessels of the skin. Thus, it is possible that deficiency may hinder developmental and physiological processes. For instance, removal of these fibulin genes in animal models was associated with multiple deformities such as Bleeding (Fibulin1), Retinopathies (Fibulin2), Macular dystrophy (Fibulin3), and defective elastic fiber formation (Fibulin5) (Kostka et al. 2001; Weigell-Weber et al. 2003; Stone et al. 1999; Yanagisawa et al. 2002). However, in the study by Tsunezumi et al., Fbln7^−/−^ mice did not show any abnormalities associated with elastic fibers or any other deformities, as seen in the absence of the rest of the members of the fibulin family. In this report, Fbln7^−/−^ pups were healthy and fertile without any known gross abnormalities indicating that Fbln7 may not be involved with elastic fiber for development (Tsunezumi et al. 2018). Further analysis of tissue-specific conditional knockout mice will provide more insight into the functionality of Fbln7 in various tissues.

### Expression and role of Fibulin7 and its fragments in pathological conditions

Since the discovery of Fbln7, multiple laboratories are making various attempts to understand its functions. Its unique tissue expression patterns, i.e., in the avascular areas of the eye, cartilage, as well as immune-privileged areas such as the placenta, have led the researchers to look into the functions of the full-length Fbln7, its fragments and shorter peptides in various pathological conditions. Moreover, very recently, overexpression of Fbln7 has also been observed in certain cancers such as glioblastoma (de Vega et al. 2019). The following paragraphs will focus on the recent findings and reports on Fbln7 with respect to pathological conditions.

#### Fbln7 and angiogenesis

In addition to cell-cell and cell-matrix (ECM) interactions in physiological processes, Fbln7 also regulates the process of angiogenesis or the formation of new blood vessels (de Vega et al. 2014). Angiogenesis is a crucial step in many pathological conditions, such as cancers (De Palma et al. 2017). Recent reports from de Vega et al. and Ikeuchi et al. highlighted that Fbln7-C binds to HUVEC cells via their β1 integrins and inhibits cell spreading, tube formation, and vessel sprouting (Ikeuchi et al. 2018; de Vega et al. 2014). Although Fbln7-C does not hinder the formation of focal complexes, it inhibits their maturation into focal adhesions, which are mediated by continuous phosphorylation of FAK and p130Cas; and sustained activation of Rac1. Interestingly, activation of Rac1 with a decreased RhoA activity leads to the formation of stellate-like morphology in HUVECs due to diminished acto-myosin contractility (de Vega et al. 2014). Although the molecular switch behind Fbln7 mediated suppression is not fully understood, its interaction with integrins and heparan sulfate receptors might likely be involved in inducing such an effect on the cell spreading and migration (Fig. 2b). In support of the antiangiogenic effect of Fbln7-C, Ikeuchi et al., using a rat corneal angiogenesis model showed that Fbln7-C binds to both vascular endothelial growth factor receptor 2 (VEGFR2) and integrin α5β1 and inhibit phosphorylation of VEGFR2 and Extracellular Signal Regulated Kinase (ERK1/2) (Fig. 2b) (Ikeuchi et al. 2018).

Peptides are reported to be the best choice for immunotherapeutics because of their low toxicity and high specificity (Rosca et al. 2011). To this end, de Vega et al. and Ikeuchi et al. have shown that one of the short peptides, fc10 generated from the globular domain of Fbln7-C mimics the anti-angiogenic properties of Fbln7-C. However, the authors were not able to test the effect of the entire globular domain due to protein aggregation (de Vega et al. 2016; Ikeuchi et al. 2020). It is hypothesized that under in vivo scenario, the presence of various angiogenic stimuli such as proteases and cathepsins results in proteolytic cleavage of Fbln7 into Fbln7-C or similar fragments, thus exposing several interacting domains, which were otherwise hidden in full-length structure (de Vega et al. 2016).

#### Fibulin7 and inflammation

Under inflammatory conditions, the interaction between ECM proteins and cell surface integrins modulates cellular functions such as cytokine production. Many fibulins have been shown to play a very significant role in inflammatory and immunological diseases, such as osteoarthritis (Mobasheri 2012; Xiang et al. 2006; Nakasaki et al. 2015; Liu et al. 2017; Fontanil et al. 2014). Due to the unique localization of Fbln7 in the immune-privileged tissues such as cornea and placenta, it is conceivable that Fbln7 or its peptides could have immunoregulatory functions. In support of this hypothesis, a recent report demonstrated that Fibulin7-Full Length (Fbln7-FL) and Fbln7-C inhibits the inflammatory properties of monocytes and macrophages, both in vitro and in vivo (Sarangi et al. 2018). The results showed that the recombinant Fbln7-FL and its C fragment proteins, bind to primary monocytes via integrins and inhibit functions such as spreading, adhesion, differentiation, and production of inflammatory mediators (Fig. 2b). Interestingly, the C terminal fragment shows a more potent activity compared to the full-length Fbln7 protein, probably due to the exposure of certain hidden interactive domains in the fragmented protein. Intravenous administration of Fbln7-C in an endotoxemia model of systemic inflammation inhibited the infiltration of both macrophages and neutrophils and reduced major histocompatibility complex (MHC) expression on the macrophages isolated from the peritoneum of lipopolysaccharide (LPS) injected mice (Sarangi et al. 2018). This study provides evidence in support of the consideration of Fbln7 and its fragments as a potential anti-inflammatory therapeutic.

#### Fibulin7 and cancers

Abnormal blood vessel formation and vascular remodeling is an essential part of cancer pathogenesis. Due to the fact that Fbln7 is expressed in endothelial cells and its C terminal fragment inhibits blood vessel formation, it is conceivable that Fbln7 may be associated with cancer pathogenesis. As per the latest human protein atlas database (https://www.proteinatlas.org) on cancers, Fbln7 is expressed in multiple types of cancers, especially in gliomas, they are highly upregulated (de Vega et al. 2019). Confirming the role of Fbln7 in cancers, de Vega et al. recently showed that Fbln7 is over-expressed by the glioblastoma tissue among astrocytic tumors, where it was primarily localized in the endothelial cells and pericytes of the glomeruloid and hypertrophied microvessels (de Vega et al. 2019). The report showed that the N-terminal sushi domain of Fbln7 interacts with angiopoietin-1 (Ang1) and inhibits the Ang1-Tie2 interaction and the subsequent phosphorylation of the Tie2 receptor. This interaction is responsible for the stabilization and normalization of blood vessels. As a result, the balancing effect of Ang1-Tie2 and Ang2-Tie2 interactions is disturbed, leading to the formation of aberrant blood vessels. The study also showed that aberrant in vitro tube formation on HUVEC and human brain vascular pericytes was inhibited by blocking or knocking down Fbln7. Also, it has been shown earlier that the C terminal fragment of Fbln7 (Fbln7-C), as well as its shorter peptide, inhibits HUVEC cell proliferation and corneal angiogenesis. Therefore, targeting both the C-terminal and N-terminal of Fbln7 protein is an essential approach to inhibit the angiogenesis by targeting the C-terminal mediated integrin based inhibition as well as N-terminal based Ang1/Tie2 axis targeting. Besides, many reports have advocated for a vital role of ECM proteins and integrin receptors in cancer progression and therapeutic interventions (Desgrosellier and Cheresh 2010; Liu et al. 2008; Wang et al. 2014). Similarly, cancer immunoediting and various cancer and immune cell interactions frame the cancer microenvironment resulting either in elimination or escape of cancer cells. Our recent data demonstrate that the administration of Fbln7-C in animals with already developed breast tumors delays the reprogramming of tumor-associated macrophages (TAM), which may be mediated by negative regulation of STAT3 pathway (Chakraborty et al. 2020). Thus, administration of Fbln7-C or endogenously expressed Fbln7 in the endothelial cells may be beneficial in modifying the functions of cancer and immune cells such as TAM and regulate cancer pathogenesis (Fig. 2b).

#### Fibulin7 in ocular disorders

Glaucoma, a chronic eye disease, involves the apoptosis of retinal ganglion cells and functional loss of the trabecular meshwork (TM) (Basu et al. 2019). It is one of the leading causes of permanent blindness affecting more than 60 million people globally (Quigley and Broman 2006). Very recently, Basu et al. reported the existence of Fbln7 in human aqueous humor (hAH) (Basu et al. 2019). The study showed that Fbln7 expression in hAH of primary angle-closure glaucoma (PACG) patients is significantly lower as compared to non-glaucomatous controls and primary open-angle glaucoma (POAG) patients. This observation established the possibility of Fbln7 to be a biomarker for PACG. The authors claimed the role of Fbln7 in the TM to be mainly structure related and not in any protective function (Fig. 2a) (Basu et al. 2019). Further studies with a larger sample size will determine the potential of Fbln7 to be a biomarker in PACG.

### Fbln7 and its bioactive fragments as potential future therapeutic

As described in the previous sections, Fbln7 has multiple modules that are structurally and functionally unique and perform a variety of interactions with different molecules (de Vega et al. 2009; de Vega et al. 2007). As described earlier in the article, a comprehensive investigation with various fragments of Fbln7 by multiple recent reports advocates for the potential of Fbln7 and its fragments and short peptides, e.g., Fbln7-C and fc-10, in alleviating angiogenesis and inflammation (de Vega et al. 2016; Sarangi et al. 2018; Ikeuchi et al. 2018). In the past, therapeutics targeting various integrin receptors was shown to be effective in many inflammatory diseases and cancers (Cox et al. 2010). Further studies are needed to understand the interacting partners for Fbln7-C or fc-10, which may be contributing to their anti-angiogenic and immunomodulatory functions. An in-depth study of this question will assist in evaluating the benefits of such reagents over integrin targeting reagents. Nonetheless, the existing data does suggest that Fbln7-C serves as a tool for controlling hyper-inflammatory responses during local and systemic inflammatory conditions by targeting immune cells and also has the potential for anti-cancer therapeutics targeting cancer cells, angiogenesis, as well as the tumor-infiltrating TAMs. (Fig. 2b) (Sarangi et al. 2018; Ikeuchi et al. 2018; Chakraborty et al. 2020). Interestingly, in contrast to the C-terminal fragments of Fbln7, the N-terminal fragment was shown to interact with Ang1 and suppress Tie2 activation in endothelial cells, ultimately resulting in aberrant blood vessel formation, which is a characteristic pathology in glioblastoma (de Vega et al. 2019). Therefore, targeted antibody or small peptide therapy against the N-terminal domain of Fbln7 may help in improving the disease prognosis. However, extensive clinical investigations are required to confirm the use of Fbln7 related therapeutics in the clinical setting.

## Conclusion

Following Fbln7’s discovery, data obtained on the expression patterns of the protein and the effects of the bioactive fragments and short peptides of Fbln7, have demonstrated an essential role of Fbln7 in physiological and pathological conditions. However, many questions still remain to be answered regarding its tissue-specific functions and the clinical applications of Fbln7-C and the shorter peptides in pathological conditions. Since Fbln7 binds to integrin receptors, a detailed analysis of its interaction with β1 integrin will definitely assist in predicting the consequences of its expression dynamics during various conditions as well as its correlation with specific signaling molecules. While in their initial discovery report, Susana et al. have demonstrated that treatment with Cathepsin-D could cleave Fbln7 in vitro, it is still not known whether bioactive fragments of Fbln7 exist under physiological or pathological conditions. This area requires an in-depth investigation. In summary, like other members of the fibulin family of proteins, evidence to date advocates for a unique function of Fbln7 in development and diseases, and further research would unravel more interesting details on the biology of this protein.

## Data Availability

Not applicable.

## Abbreviations

Fbln7: Fibulin7
EGF: Epidermal Growth Factor
cbEGF: Calcium-binding EGF
CCP: Complement Control Protein
DSPP: Dentin sialophosphoprotein
HUVEC: Human Umbilical Vein Endothelial Cells
VEGFR2: Vascular Endothelial Growth Factor Receptor 2
ERK1/2: Extracellular Signal Regulated Kinase
HBVP: Human Brain Vascular Pericytes
hAH: Human Aqueous Humor
PACG: Primary Angle Closure Glaucoma
POAG: Primary Open Angle Glaucoma
MMP: Matrix Metalloproteinase
TAM: Tumor Associated Macrophages
LPS: Lipopolysaccharide
